# T-cell and serological responses to Erp, an exported *Mycobacterium tuberculosis *protein, in tuberculosis patients and healthy individuals

**DOI:** 10.1186/1471-2334-7-83

**Published:** 2007-07-26

**Authors:** Valérie Martinez, Guislaine Carcelain, Edgar Badell, Marc Jouan, Isabelle Mauger, Pierre Sellier, Chantal Truffot, François Bricaire, Sandra M Arend, Tom Ottenhoff, Brigitte Autran, Brigitte Gicquel

**Affiliations:** 1 Laboratoire d'Immunologie Cellulaire, INSERM U543, Hôpital Pitié-Salpêtrière, 47-83, Boulevard de l'Hôpital, 75651 Paris Cedex 13, France; 2 Unité de Génétique des Mycobactéries, Institut Pasteur, 25, rue du Docteur ROUX, 75015 Paris, France; 3 Service de Médecine Interne, Hôpital Lariboisière, 2, Rue Ambroise Paré, 75010 Paris, France; 4 Laboratoire de Bactériologie, Hôpital Pitié-Salpêtrière, 47-83, Boulevard de l'Hôpital, 75651 Paris Cedex 13, France; 5 Service des Maladies Infectieuses et Tropicales, Hôpital Pitié-Salpêtrière, 47-83, Boulevard de l'Hôpital, 75651 Paris Cedex 13, France; 6 Department of Infectious Diseases and Department of Immunohematology and Blood Transfusion, Leiden University Medical Center, Leiden, Netherlands

## Abstract

**Background:**

The identification of antigens able to differentiate tuberculosis (TB) disease from TB infection would be valuable. Cellular and humoral immune responses to Erp (Exported repetitive protein) – a recently identified *M. tuberculosis *protein – have not yet been investigated in humans and may contribute to this aim.

**Methods:**

We analyzed the cellular and humoral immune responses to Erp, ESAT-6, Ag85B and PPD in TB patients, in BCG^+ ^individuals without infection, BCG^+ ^individuals with latent TB infection (LTBI) and BCG^- ^controls. We used lymphoproliferation, ELISpot IFN-γ, cytokine production assays and detection of specific human antibodies against recombinant *M. tuberculosis *proteins.

**Results:**

We included 22 TB patients, 9 BCG^+ ^individuals without TB infection, 7 LTBI and 7 BCG^- ^controls. Erp-specific T cell counts were higher in LTBI than in the other groups. Erp-specific T cell counts were higher in LTBI subjects than TB patients (median positive frequency of 211 SFC/10^6 ^PBMC (range 118–2000) for LTBI subjects compared to 80 SFC/10^6 ^PBMC (range 50–191), p = 0.019); responses to PPD and ESAT-6 antigens did not differ between these groups. IFN-γ secretion after Erp stimulation differed between TB patients and LTBI subjects (p = 0.02). Moreover, LTBI subjects but not TB patients or healthy subjects produced IgG3 against Erp.

**Conclusion:**

The frequencies of IFN-γ-producing specific T cells, the IFN-γ secretion and the production of IgG3 after Erp stimulation are higher in LTBI subjects than in TB patients, whereas PPD and ESAT-6 are not.

## Background

Tuberculosis (TB) remains a major public health problem. It is estimated that one third of the world's population has latent *Mycobacterium tuberculosis *infection [1]. Vaccination with Bacillus of Calmette-Guérin (BCG) confers only partial protection. The protective immune responses elicited by tuberculosis infection or vaccination with BCG in non endemic countries remain poorly understood. After infection, activated macrophages produce interleukin (IL)-12, which in turn stimulates CD4 T cells to produce Th1 cytokines (IFN-γ and TNF-α), thus linking the innate and adaptive immune responses [2,3]. Th1 cellular responses are essential for effective protection against TB in several animal models. Although the immune responses induced by TB are generally able to contain the pathogen, they are unable to eliminate it, and 5 to 10% of immunocompetent individuals develop tuberculosis [2,4].

Many studies have investigated immune responses in TB patients and healthy contacts. As BCG vaccination is widely used in countries with a high incidence of tuberculosis, we decided to use a new *M. tuberculosis *antigen, Erp, to analyze cellular and humoral immune responses in TB patients and in healthy individuals, including both those who had or had not been vaccinated with BCG. In infected humans and animal models, both surface and secreted antigens such as ESAT-6, Ag85B antigens and heparin-binding hemagglutinin adhesin, have been identified as inducers of Th1 CD4 cellular immune responses [5-9]. Erp is (Exported repetitive protein), is an antigen exported by *Mycobacterium *species [10-12]. The Erp gene encodes a cell-surface component with a repetitive structure [11,13,14]. Erp is present in all strains of mycobacteria, including BCG strains, but its sequence varies (12). It is required for the survival and multiplication of mycobacteria [10,15]. Homologs of this antigen are immunodominant in both lepromatous leprosy and bovine tuberculosis [15,16]. Cellular and humoral immune responses against this antigen have not been investigated in humans and only animal studies are available.

ESAT-6 is an early-secreted antigen, encoded by the RD-1 genetic region. This region is absent, presumably deleted, from *M. bovis *BCG strains and in environmental mycobacteria [17-19]. T cell responses to ESAT-6 differ between TB patients and healthy BCG-vaccinated controls and can be used to identify symptom-free individuals recently exposed to *M. tuberculosis *[20-25]. Ag85B, which belongs to the 85 complex including 85A, B and C antigens, is involved in the final stages of the cell wall synthesis [26,27]. This antigen complex induces strong Th1 cellular immune responses in PPD-positive healthy individuals, but weak responses in 50% of TB patients [28,29]. Moreover, inclusion of Ag85A or Ag85B in a protein or DNA vaccine confers significant protection against tuberculosis in mice [30,31]. We studied and compared specific cellular and humoral immune responses to Erp, an antigen of latency, with those to ESAT-6, Ag85B, 2 antigens involved in the virulence of the mycobacteria and PPD as comparison in TB patients and in individuals vaccinated (healthy and latent TB) or not vaccinated with BCG.

Immune responses against Erp were detected in TB patients and in BCG^+ ^individuals both without infection and with latent TB infection (LTBI). The frequencies of IFN-γ-producing specific T cells, the IFN-γ secretion and the production of IgG3 were higher in BCG^+ ^subjects with LTBI after Erp stimulation. This pilot study suggests that Erp-specific responses may be a valuable marker to distinguish LTBI from TB subjects, and should be evaluated in larger studies.

## Methods

### Patients

This prospective study was carried out in the Department of Infectious Diseases of Pitié-Salpêtrière Hospital and in the Department of Internal Medicine of Lariboisière Hospital, Paris, France, between February 2001 and February 2002. The study protocol was approved by the institutional review board of Pitié-Salpêtrière Hospital and was carried out in accordance with the Declaration of Helsinki.

We recruited twenty-two TB patients, according to the following criteria: ≥18 years old, and with microbiologically confirmed TB disease; patients who had already begun treatment were excluded from the study. All subjects were counselled about HIV testing and provided written informed consent.

Sixteen BCG-vaccinated individuals (BCG^+^) were enrolled, according to the following inclusion criteria: ≥18 years old, with prior BCG vaccination and no reported or known history of TB infection or disease. This group was divided into two sub-groups: those giving at least one positive response to ESAT-6 in at least one of the three tests used (lymphoproliferation, ELISpot IFN-γ, cytokine production assays) and those scoring negative in all tests. Nine subjects gave no positive responses and were considered as uninfected (BCG^+ ^without infection group) and seven displayed positive responses to ESAT-6 and were considered to have latent TB infection (LTBI).

For comparison, seven healthy non-BCG-vaccinated (BCG^-^) individuals were enrolled, according to the following criteria: ≥18 years old, no prior BCG vaccination and no history of TB exposure or infection. This last control group was recruited by the Departments of Infectious Diseases, of Immunohematology and Blood Transfusion of Leiden University Medical Center (Leiden, Netherlands).

### Preparation of peripheral blood mononuclear cells

Venous blood (40 ml) from each participant was collected into heparin. Peripheral blood mononuclear cells (PBMC) were prepared by Ficoll-Hypaque centrifugation (Eurobio) of the blood.

### CD4+ T-cell purification

CD4^+ ^T cells were purified from PBMC by magnetic separation, using a Dynabeads kit according to the manufacturer's instructions (Dynabeads, Dynal, Oslo, Norway). The purity of the CD8-depleted cells was analyzed by flow cytometry and exceeded 95%. CD8 cells accounted for less than 5% of the remaining cells.

### Antigens

A purified protein derivative of *Mycobacterium tuberculosis *(PPD) (Statens Serum Institute, Copenhagen, Denmark) was used at a concentration of 1 μg/ml as is routine in our laboratory. ESAT-6 was a gift from the Statens Serum Institute (Copenhagen, Denmark) and was used at a concentration of 1 μg/ml. Antigen Ag85B was obtained from Lionex Diagnostics and Therapeutics GmbH (Braunschweig, Germany) and was used at a concentration of 2 μg/ml. Erp produced as a recombinant antigen in *Escherichia coli *M15, purified by Aventis Pasteur SA (Marcy L'Etoile, France), was used at a concentration of 0.5 μg/ml. Antigen preparations were tested for LPS contamination: ESAT-6 was free of LPS, and Ag85B and Erp contained less than 100 EU/mg of endotoxin. Concentrations of ESAT-6, Erp and Ag85B were defined by testing a large dilution series of antigen concentrations in a population of tuberculosis patients (n = 12) and the optimal concentration of each antigen was selected.

### Evaluation of specific T-cell responses

#### Lymphoproliferation assay

The lymphoproliferation assay was carried out with freshly isolated PBMC from TB patients and BCG^+ ^controls. PBMC (1.5 × 10^5^) were cultured in 200 μl of complete medium in round-bottomed microtiter tissue culture plates (TPP^®^, Switzerland), with or without each of the antigens. We assessed the stimulation activity of all antigens. Phytohemagglutin was used as a positive control (PHA, Murex, Paris, France), at a concentration of 0.5 μg/ml. Medium alone was used as a negative control. Cells were cultured in complete medium (RPMI 1640 supplemented with 1% essential amino acids, 1% sodium pyruvate, 25 × 10^3 ^U penicillin and 25 × 10^3 ^μg streptomycin) (GibcoBRL), together with 20% pooled, HIV-free, male human AB serum (AbCys, Paris, France). Cells were incubated alone or with one of the antigens, for 6 days at 37°C, in a humidified atmosphere containing 5% CO_2_. Cells were then labeled with tritiated thymidine (CEA, Saclay, France) on day 6. Cells were harvested on fiberglass paper, using a cell harvester (Printed Filtermat A, Wallac Oy, Finland), and the incorporated radioactivity was measured in a liquid scintillation counter (Microbeta™ Plus, Wallac). Mean counts per minute for triplicate cultures and a stimulation index (SI) were obtained for each patient. The SI was the ratio of mean counts per minute in the presence of antigen to mean counts per minute in medium alone. Antigen-specific proliferative responses were considered to be positive if SI ≥ 3.

#### ELISpot-IFN-γ assay

The ELISpot IFN-γ assay was carried out with freshly prepared PBMC from TB patients and BCG^+ ^subjects and with frozen cells from BCG^- ^individuals.

The anti-human IFN-γ capture Mab (Diaclone) was used to coat 96-well polyvinylidene difluoride plates (Millipore, Molsheim, France), by incubation overnight at 4°C. Each of the various antigens (Erp, ESAT-6, Ag85B and PPD) was added to three wells (triplicate), at the concentrations indicated above. PBMC were cultured in complete medium (see above) supplemented with fetal calf serum (10%, Gibco, France). Negative controls consisted of cells cultured with medium alone and positive controls consisted of cells cultured with PHA. PBMC (1.5 × 10^5 ^per well corresponding to the number of cells inducing optimal responses to these antigens) were incubated for 40 hours at 37°C in an atmosphere containing 5% CO_2_, as previously described [32]. The plates were washed and a biotinylated anti-IFN-γ Mab (Diaclone) was added, followed by streptavidin-alkaline phosphatase conjugate (Amersham, Les Ulis, France) and chromogen substrates (5-bromo-4-chloro-3-indolyl phosphate toluidine and nitroblue tetrazolium) (Sigma-Aldrich, St. Quentin, France). Plates were dried and the colored spots were counted using an automated ELISpot reader (Zeiss, Jena, Germany). The numbers of specific T cells are expressed as spot-forming cells per million PBMC (SFC/10^6 ^PBMC), calculated after subtracting the mean values obtained without antigen for each sample. Responses were considered positive if the test well contained at least 5 IFN-γ SFC more than negative control wells.

#### Cytokine production

Cytokine production was assessed in freshly prepared cells from TB patients and BCG^+ ^controls. Freshly prepared PBMC (1.5 × 10^5^) were cultured in 96-well flat-bottomed plates (TPP^®^, Switzerland) with the antigens Erp, ESAT-6, Ag85B and PPD. The negative control consisted of medium alone and the positive control included anti-CD3 (2.5 μg/ml) and anti-CD28 antibodies (5 μg/ml; Immunotech, Coulter, France). Supernatants (150 μl) were collected after 48 hours and frozen at -20°C. The frozen supernatants were thawed at room temperature and cytokine concentrations were determined, using a commercial kit designed to detect multiple human Th1/Th2 cytokines (IFN-γ, TNF-α, IL-2, IL-4, IL-5 and IL-10) in a single sample, in accordance with the manufacturer's instructions (Cytometric Bead Array Kit, BD Pharmingen). The Cytometric Bead Array (CBA) is a flow cytometric-based assay for the detection of human cytokines from cell supernatants or serum samples. The kit uses a set of beads coated with cytokine-specific antibodies which serves as a capture surface for cytokines; we used the 6 bead kit, which includes beads specific for IFN-γ, TNF-α, IL-2, IL-10, IL-4, IL-5. Each cytokine-specific set of beads is assigned a discrete fluorescence intensity that can be resolved on the FL-3 channel of a flow cytometer. The advantage of using the CBA assay is that it is an accurate, sensitive and quantitative method for measuring cytokine levels although requiring only small volumes for analysis.

The detection limit of the assay was 50 pg/ml. The values obtained for the negative control (unstimulated) wells were subtracted from those obtained with stimulated cells and the results are expressed in pg/ml.

#### Elisa test for detection of specific human antibodies against recombinant M. tuberculosis proteins

Serum samples from a subgroup of 15 of the 22 TB patients, seven BCG^+ ^subjects and six BCG^- ^controls were available for testing. Dynatech^® ^96-well flat-bottomed plates (Dynex Technologie, France) were coated by incubation overnight with 50 μl/well of the recombinant proteins (Erp, ESAT-6, Ag85B) at a concentration of 3 μg/ml in phosphate-buffered saline (PBS). PBS was used as a negative control. Plates were blocked by incubation with 3% bovine serum albumin (BSA) (Prolabo, Fontenay-sous-Bois, France) in PBS, for 1 hour at 37°C. Serum samples, diluted 1:100 in 1.5% BSA, 0.1% Tween 20 in PBS, were incubated for 3 hours at 37°C. The specific antibody (Ab) response was then assessed by incubating with alkaline phosphatase-conjugated mouse anti-human IgG1, IgG2, IgG3 and IgG4 antibodies (Promega, Charbonnières, France) diluted 1:4000 in 1.5% BSA, 0.1% Tween 20 in PBS, for 1 hour at 37°C. The plates were washed with 0.1% Tween 20 in PBS and the colorimetric reaction was developed with p-nitrophenylphosphate (Sigma, St Louis, MO) used as the substrate. Absorbance at 405 nm was determined with a Labsystems Multiskan RC. Results were expressed in optical density units after subtraction of the values obtained with PBS and considered as positive above 0.1 OD.

### Statistical analysis

Comparisons between TB patients, BCG^+ ^and/or BCG^- ^controls were performed using non parametric tests as appropriate. Data were analyzed with StatView F-5.0 software (1992–1998, SAS Institute Inc; California).

## Results

### Patients' characteristics

All subjects, after obtention of informed consent, were tested negative for human immunodeficiency virus (HIV) and none were on immunosuppressive therapy.

The median age of the TB patients (17 men and 5 women) was 38 years (range 19–74). Most patients were Caucasian (68.2%), and the others were African or Asian. Proof of BCG vaccination was available for 8 of the 22 TB patients, and the other 14 patients were born in countries in which BCG vaccination is mandatory. Thus, it is likely that all the TB patients had been vaccinated with BCG. Six of the 22 TB patients had a history of TB disease, with non optimal treatment due to a lack of compliance in five patients and infection with a multidrug-resistant strain in one patient. The localization of the TB disease was pulmonary (n = 14), extra-pulmonary (n = 6) or both (n = 2). Blood samples were obtained before the initiation of specific treatment for all patients.

BCG^+ ^vaccinated subjects were divided into a BCG^+ ^without infection group (6 men and 3 women) and LTBI group (4 men and 3 women) according to their responses to ESAT-6. The median ages for these groups were 34 years (range 19–73) and 28 years (range 19–50) respectively. All individuals except one in each group were Caucasian and the exceptions were of African origin. All BCG^+ ^subjects had been vaccinated with BCG during childhood. No Mantoux test was performed in the BCG^+ ^group.

The median age in the BCG^- ^group (3 men and 4 women) was 27 years (range 20–42). All individuals were Caucasian. None of the BCG^- ^subjects had a history of TB disease or known contact with TB patients.

None of our subjects included in this study was anergic, all of them have positive responses to mitogen used as positive control in the different assays.

### Specific responses in the lymphoproliferation assay

**Figure 1:**
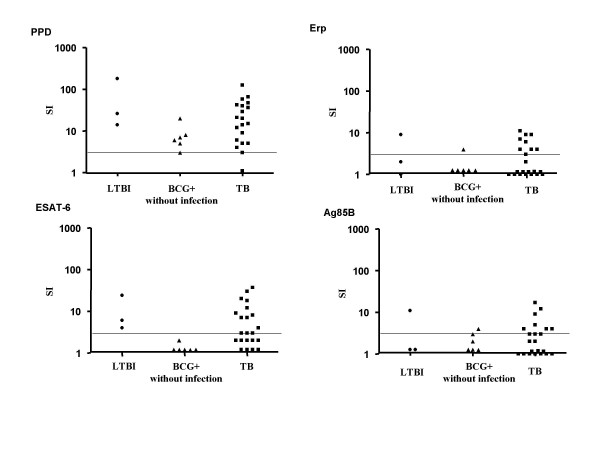
Proliferative responses to the specific mycobacterial antigens, PPD, Erp, ESAT-6 and Ag85B were evaluated in 3 groups: BCG^+ ^subjects without infection, with latent TB infection (LTBI) and in TB patients (TB), measured by lymphoproliferation assay. A logarithmic scale is used and horizontal bars indicate positive values.

Lymphoproliferation assays were carried out for the 20 of the 22 TB patients for who sufficient cells were available and for nine BCG^+ ^subjects including six BCG^+ ^without infection and three with LTBI (Figure 1). BCG^- ^subjects were not tested, as fresh cells were not available. Most TB patients (19/20) and all (9/9) BCG^+ ^subjects (LTBI and without infection subjects) displayed positive proliferative responses with the PPD antigen. The median SI was 17.5 (range 1–125), 6.5 (range 3–20) and 26 (range 14–180) for TB, BCG^+ ^without infection and LTBI groups, respectively.

A lower percentage of subjects from both groups displayed positive proliferative responses to the specific mycobacterial antigens. For Erp, 41% (9/22) of the TB, 17% (1/6) of the BCG^+ ^without infection and 1/3 of the BCG^+ ^with LTBI groups displayed a positive proliferative response, with an overall low median SI of 1 (range 1–11), 1 (range 1–4) and 2 (range 1–9), respectively. For ESAT-6, 59% (13/22) of the TB, none (0/6) of the BCG^+ ^without infection and all (3/3) of the LTBI groups displayed a positive proliferative response, with an overall median SI of 3 (range 1–37), 1 (range 1–2) and 6 (range 4–24), respectively. For Ag85B, 45% (10/22) of the TB, 33% (2/6) of the BCG^+ ^without infection and 33% (1/3) of the LTBI groups displayed a positive proliferative response, with an overall low median SI of 2 (range 1–17), 1.5 (range 1–4) and 1 (range 1–11), respectively. So the responses to the 4 antigens appeared to be very similar for the 3 groups of patients. However, although LTBI patients responded to ESAT-6, BCG^+ ^patients without infection did not, as would be expected from the definition of the two groups. Finally, we observed no difference in immune responses, as evaluated by lymphoproliferation assay, according to the site of TB infection.

### IFN-γ responses detected by ELISpot assays depend on TB infection or disease status

The ELISpot IFN-γ assay was used to quantify antigen-specific CD4 T cells in 21 of the 22 TB patients, nine of the BCG^+ ^without infection group, seven of the LTBI and seven of the BCG^- ^controls. The ELISpot IFN-γ assay was carried out with freshly prepared PBMC from TB patients and BCG^+ ^subjects and with frozen cells from BCG^- ^individuals, after preliminary analysis to confirm that there was no difference between the results obtained with freshly prepared and frozen cells from BCG^+ ^controls (n = 5): no significant difference was observed for Erp, ESAT-6, Ag85B, PPD or PHA (p > 0.05) between the two cell preparations.

**Figure 2:**
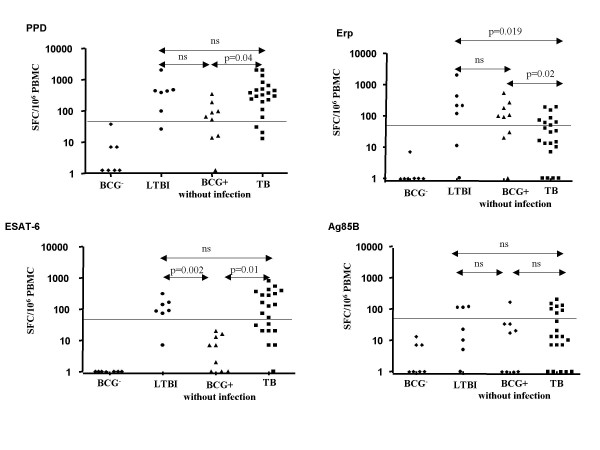
Numbers of antigen-specific T cells in the 4 groups:non BCG-vaccinated controls (BCG^-^), BCG^+ ^subjects without infection and with latent TB infection (LTBI), and in TB patients (TB), measured by ELISpot IFN-γ assay after stimulation with PPD, Erp, ESAT-6 and Ag85B. A logarithmic scale is used and horizontal bars indicate positive values.

We analyzed total PBMC and purified CD4 T cells for the first five TB patients. Similar numbers of antigen-specific T cells were obtained in the two assays. We therefore carried out subsequent experiments with total PBMC, given that the results accurately reflected CD4 T-cell responses and the cutoff point was at least five IFN-γ SFC more than in negative control wells. The results are summarized in Figure 2.

BCG^- ^controls displayed no IFN-γ cellular response to the mycobacterial antigens Erp, ESAT-6, Ag85B and PPD as expected, despite displaying normal responses to PHA.

TB patients developed IFN-γ-specific cellular responses, directed against PPD and ESAT-6 in particular. A specific T-cell response to PPD was detected in 86% (18/21) TB patients, with a median positive frequency of375 SFC/10^6 ^PBMC (range 62–2000). Sixty-two percent (13/21) of TB patients displayed T-cell responses to ESAT-6, with a median positive frequency of280 SFC/10^6 ^PBMC range 53–799). Fewer TB patients displayed specific cellular responses to Erp (38%, 8/21) and Ag85B (33%, 7/21), with a median positive frequency of 80 SFC/10^6 ^PBMC (range 50–191) and120 SFC/10^6 ^PBMC (range 73–200), respectively.

LTBI subjects developed specific cellular responses, directed against PPD, Erp and ESAT-6 in particular: 85.7% (6/7) developed a PPD-specific T-cell responses (median positive frequency434 SFC/10^6 ^PBMC (range 99–2000)) and 71% (5/7) developed Erp-specific T-cell responses (median positive frequency 211 SFC/10^6 ^PBMC (range 118–2000)). ESAT-6-specific CD4 Th1 cell responses were observed in 86% (6/7) of LTBI subjects (median positive frequency 115 SFC/10^6 ^PBMC (range 73–310)), but only 43% (3/7) had Ag85B-specific CD4 Th1 responses (median positive frequency112 SFC/10^6 ^PBMC (range 112–119)).

BCG^+ ^subjects without infection developed specific cellular responses, directed only against PPD and Erp: 67% (6/9) developed a PPD-specific T-cell responses (median positive frequency 95 SFC/10^6 ^PBMC (range 53–350)) and 67% (6/9) developed Erp-specific T-cell responses (median positive frequency142 SFC/10^6 ^PBMC (range 92–541)). Moreover, one out of 9 subjects had Ag85B-specific CD4 Th1 responses (165 SFC/10^6 ^PBMC). As expected, none of the BCG^+ ^subjects without infection gave ESAT-6-specific CD4 Th1 cell responses.

Numbers of PPD-specific T cells were similar for the three groups of subjects (TB, BCG^+ ^without infection group, LTBI subjects) and higher than those BCG^- ^group (p < 0.01). ESAT-6-specific T cells were similar in the TB and LTBI groups, and significantly more numerous than in the other groups (p ≤ 0.01). Only numbers of Erp-specific T cells were significantly higher in BCG^+ ^individuals without infection or with LTBI than in TB patients (p ≤ 0.02). This difference between LTBI and TB patients observed after Erp stimulation was not found for PPD and ESAT-6 antigens.

### Cytokine production

**Figure 3:**
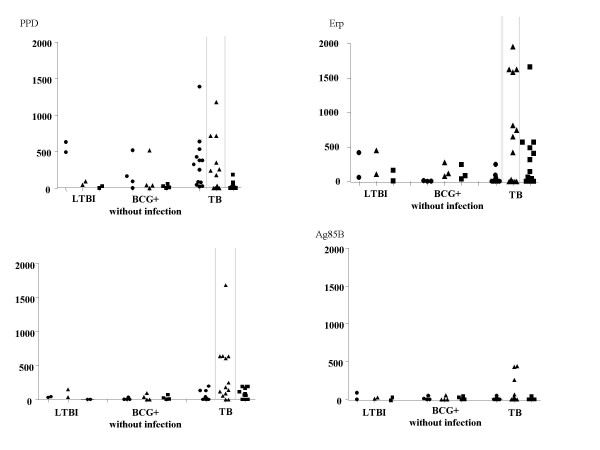
Cytokine productions (IFN-γ or ●, TNF-α or ▲ and IL-10 or ■) were represented for the 3 groups: BCG^+ ^subjects without infection, with latent TB infection (LTBI) and in TB patients (TB).

We evaluated the production of Th1/Th2 cytokines in a subgroup of 13 TB patients and six BCG^+ ^subjects (4 BCG^+ ^individuals without infection and 2 with LTBI) for whom cells were available. BCG^- ^subjects were not tested, because cells were not available. The Th1 (IFN-γ, TNF-α, IL-2) and Th2 (IL-4, IL-5, IL-10) cytokines present in the supernatants of stimulated cells were quantified by flow cytometry and the results are presented in Figure 3.

IFN-γ, TNF-α and IL-10 were detected after stimulation with each of the antigens tested: Erp, ESAT-6, Ag85B and PPD. By contrast, IL-2, IL-4 and IL-5 were not detected following stimulation with any of the antigens (data not shown).

In TB patients, IFN-γ, TNF-α and IL-10 productions were detected after stimulation with PPD (median 325, range 22–1389; median 179, range 0–1178; median 12, range 0–182 respectively), ESAT-6 (median 13, range 0–4597; median 179, range 0–1679; median 65, range 0–191 respectively) and Erp (median 11, range 0–245; median 652, range 0–1961; median 228, range 0–1662 respectively).

In the LTBI group, IFN-γ and TNF-α productions were also detected after stimulation with PPD, Erp and with ESAT-6 stimulation for the 2 patients tested. IL-10 production was very low.

In three of the four BCG^+ ^individuals without infection, IFN-γ and TNF-α production were mainly detected only after PPD stimulation. No IFN-γ or TNF-α production was detected after stimulation with ESAT-6. IL-10 and TNF-α were detected after Erp stimulation.

Poor production of cytokine was detected after stimulation with Ag85B in the 3 groups of patients.

#### Humoral responses

**Figure 4:**
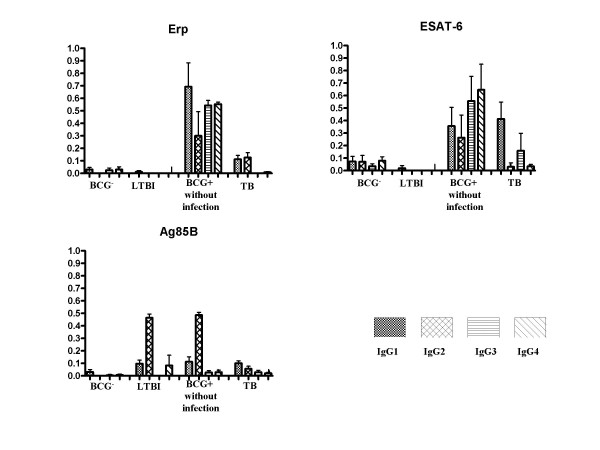
Subtypes of IgG were represented in column bar after Erp, ESAT-6 and Ag85B stimulation in the 4 groups: non BCG-vaccinated controls (BCG^-^), BCG^+ ^subjects without infection, with latent TB infection (LTBI) and in TB patients (TB) in the order: IgG1, IgG2, IgG3, IgG4.

We tested the available serum samples from the individuals included in the study (15 TB patients, 4 BCG^+ ^subjects without infection and 3 LTBI subjects) for the presence of subclasses of IgG antibodies against Erp, ESAT-6 and Ag85B (Figure 4). Specific antibody levels were highly heterogeneous in all groups, resulting in high standard deviations.

For the BCG^- ^group, levels of specific IgG subclasses were below this threshold for all antigens.

TB patients developed specific IgG1 (n = 8) and IgG2 (n = 7) against Erp and IgG1 (n = 11) and IgG3 (n = 2) against ESAT-6 (Figure 4).

All LTBI individuals produced at least three of IgG subtypes IgG1, IgG2, IgG3 and IgG4 after Erp or ESAT-6 stimulation. In the BCG^+ ^group, no IgG were detected against Erp, ESAT-6.

We observed high production of IgG3 against Erp in LTBI subjects but IgG3 was not produced by TB patients or by BCG^+ ^individuals without infection. The profile of responses to Erp and ESAT-6 antigens was consistent with the findings of the assays evaluating cellular immunity.

## Discussion

We studied cellular and humoral immune responses to Erp, a recently identified *M. tuberculosis *protein and three mycobacterial antigens: PPD, ESAT-6 and Ag85B.

In our study, most TB patients responded to ESAT6. This is consistent with published studies [9,33,34] In which 55 to 95% of TB patients displayed an ESAT-6-specific Th1 response, as evaluated by lymphoproliferation assays or by determining IFN-γ concentration in the supernatant of stimulated cells [22,33]. ESAT-6, a RD-1-encoded *M. tuberculosis*-specific antigen, is an accurate marker of *M. tuberculosis *infection; it discriminates against BCG because the RD-1 region is deleted from such strains [20-22,24,35]. It can therefore be used to differentiate between individuals with and without TB infection, regardless of BCG vaccination status [36-39]. Nevertheless, 43% of BCG-vaccinated subjects responded to ESAT-6 in our study. There are several possible explanations for this result: exposure to *M. tuberculosis *is not implausible because 12.5% of the participants originated from Africa or Asia and 31% were hospital workers. Thus, these findings may be the consequence of cases of latent TB infection despite no reported previous TB contact or exposure to environmental mycobacteria in France or during visits to countries in which these bacteria are highly prevalent. These observations led us to distinguish between BCG^+ ^individuals without infection (ESAT-6^-^) and with LTBI (ESAT-6^+^) in our analysis.

We also analyzed immune responses to Ag85B, a mycobacterial antigen involved in the final stages of cell wall synthesis [26,40,41]. Subjects in all groups displayed weak humoral and cellular immune responses after Ag85B antigen stimulation, consistent with the findings of previous studies [36,42-44].

Erp is an exported protein encoding a cell-surface component [11,13,14], and is present in all strains of mycobacteria, including BCG strains. Erp is crucial for the survival and multiplication of mycobacteria [10,15]. In this study, we demonstrated that Erp induced cellular and humoral responses in BCG^+ ^subjects without infection, in LTBI subjects and in TB patients. The Erp antigen has previously only been studied in macrophage cell lines and *in vivo *in mouse models. This study is the first to evaluate human cellular and humoral immune responses to Erp in TB patients and in individuals vaccinated and not vaccinated with BCG. We show that LTBI subjects had more Erp-specific IFN-γ-producing cells than TB patients and Erp-specific IgG3 were present only in infected but non-ill BCG^+ ^subjects corresponding to latent TB infection. These results are consistent with previous findings concerning the production of IFN-γ by activated T cells, which is required for the regulation of isotype switching during B-cell development [45-47]. IFN-γ regulates the isotype specificity of switching and can induce switching to IgG3 [47]. Furthermore, the orientation of the response toward the IgG3 subclass is surface antigen-specific [48-50]. Erp is a surface antigen, whereas ESAT-6 is a secreted antigen, and this explains the detection of IgG3 after Erp stimulation. An association has been found between antibody isotype, cytokine profile and pathogenesis in some infectious diseases, including leprosy and malaria, but never in tuberculosis [51-54]. Moreover, T cell-derived cytokines, such as IL-10, have been reported to be involved in the regulation of switch factors for IgG3 [55]. The significance of IgG3 specificity in infectious diseases remains unclear. Functionally, IgG3 is considered to be the most effective subclass for activating the complement pathway and mediates cell lysis by monocytes and Fc receptor-bearing lymphocytes. In a study of severe malaria, Sarthou et al. demonstrated that only *P. falciparum-*specific IgG3 levels were positively correlated with survival [56]. So, in light of published data and our results, IgG3 against Erp could be used as a surrogate marker of protection against TB. Moreover, Erp, an exported surface antigen but not a secreted antigen, was not directly associated with virulence but may be implicated in latency, as in our study. This antigen is potentially useful for the diagnosis of tuberculosis disease versus latent infection and this possibility should be tested in a very large series of patients.

## Conclusion

Our results show that Erp induces T-cell or humoral responses in TB patients and in BCG^+ ^subjects without infection and with LTBI. The responses against this antigen, unlike those against PPD and ESAT-6, are higher in LTBI individuals than in TB patients. The response to Erp may therefore be useful for the diagnosis of TB discriminating between disease and infection with *M. tuberculosis*. The choice of antituberculous therapy and of therapy duration depends on this distinction between infection and disease. This interesting possibility arising from our pilot study needs to be tested in studies with a large number of patients to confirm the possible contribution of Erp to the diagnosis of latent TB infection.

## Competing interests

All authors declare that they have no competing financial interests and also no other non-financial (political, personal, religious, ideological, academic, intellectual, commercial or any other) competing interests that may cause them embarrassment were they to become public after the publication of the manuscript.

## Authors' contributions

VM: participated in the study design, coordination, performed the assays and drafted the manuscript.

GC: participated in its design and coordination and helped to draft the manuscript.

EB: participated in its design and coordination and helped to draft the manuscript.

MJ: conceived of the study, and participated in its design.

IM: helped perform the assays.

PS: provided patients and contributed substantially to the acquisition of data

CT: provided patients and contributed substantially to the acquisition of data

FB: provided patients and contributed substantially to the acquisition of data

SA: kindly provided the samples from the BCG^- ^group and helped to draft the manuscript.

TO: kindly provided the samples from the BCG^- ^group and helped to draft the manuscript.

BA: participated in its design and coordination and helped to draft the manuscript.

BG: participated in its design and coordination and helped to draft the manuscript.

All authors read and approved the final manuscript.

## Pre-publication history

The pre-publication history for this paper can be accessed here:


